# Antegrade Ureteral Stenting in Pediatric Patients: Introducing a Novel Ureteral Morphological Classification and a New Perspective on Functional Success

**DOI:** 10.3390/jcm14010246

**Published:** 2025-01-03

**Authors:** Mustafa Mazıcan, Ismail Karluka, Abdulkerim Temiz, Cagatay Andic

**Affiliations:** 1 Interventional Radiology Department, Adana Dr. Turgut Noyan Application and Research Center, Başkent University, 01250 Adana, Turkey; ismailkarluka@baskent.edu.tr (I.K.); candic@baskent.edu.tr (C.A.); 2 Pediatric Surgery Department, Adana Dr. Turgut Noyan Application and Research Center, Başkent University, 01250 Adana, Turkey; abdulkerimtemiz@baskent.edu.tr

**Keywords:** pediatric urology, antegrade ureteral stenting, ureteral morphology, functional success, hydronephrosis, double-J stent

## Abstract

**Background/Objectives**: The objective of the current research is to assess the benefits that come with antegrade ureteral stenting coupled with imaging techniques in children and also provide a new classification based on the ureter’s morphological elements. **Methods**: Between 2011 and 2024, 107 antegrade stent placement procedures performed in 71 pediatric patients aged 0–12 years who could not undergo retrograde double-J stent placement were retrospectively analyzed. According to the morphologic structure of the ureter, four categories were classified as normal, straight/slightly angled, S-shaped, and spiral-shaped. Functional success was evaluated by comparing the results of ultrasonography and Tc-99m MAG3 scintigraphy before and after the procedure. **Results**: Technical success rate was 99.1% and functional success rate was 84.1%. Intra-procedural complication rate was 5.6% and post-procedural complication rate was 39.3%. According to morphologic classification, the functional success rate was lowest in spiral-shaped ureters with 79.5%, but this difference was not statistically significant (*p* = 0.775). There was no significant correlation between stent diameter, balloon dilatation, and degree of hydronephrosis and functional success. **Conclusions**: Antegrade ureteral stent placement is a safe method with high technical success and acceptable complication rates in pediatric patients. The developed ureteral morphologic classification may guide clinical practice.

## 1. Introduction

In pediatric urology, ureteral stenting is a widely used method for managing various urinary tract conditions [1]. However, retrograde stent placement may not be feasible in some pediatric patients due to anatomical or technical challenges [2]. In such cases, imaging-guided antegrade stenting serves as an effective alternative [3], offering high success rates and minimal complications with the use of ultrasound and fluoroscopy [4,5,6]. Percutaneous antegrade ureteral stenting (PAUS) is particularly valuable in children with urinary obstructions where retrograde approaches are unsuitable or definitive surgical treatment is not immediately feasible [7].

Imaging-guided interventions, such as percutaneous nephrostomy (PCN) and antegrade ureteral stent placement, are essential tools in treating obstructive urinary conditions [8]. Functional outcomes are frequently assessed using Tc-99m MAG3 scintigraphy and ultrasound, which evaluate kidney function and obstruction relief [8]. These techniques allow interventional radiology to offer safe and reliable solutions tailored to the unique needs of pediatric patients [8].

PAUS has been shown to be a reliable option for ensuring urinary drainage in a variety of conditions, including ureteropelvic junction obstruction (UPJO), vesicoureteral reflux (VUR), and other congenital or acquired causes of urinary obstruction [7]. Technical success rates for PAUS in pediatric patients are reported to be as high as 97.6%, with clinical success rates of 90% [7]. Although minor complications, such as urinary tract infections, stent migration, and early occlusion, may occur, these issues are generally manageable and do not compromise the overall effectiveness of the procedure [7]. Balloon dilation is often performed to address severe ureteral stenosis and has been successfully integrated into PAUS protocols, ensuring high technical success even in challenging cases [7].

Hydronephrosis, characterized by the dilation of the renal pelvis and calyces due to urine flow obstruction, is one of the most common abnormalities encountered in pediatric urology [9]. While many cases resolve spontaneously, certain conditions like UPJO and primary obstructive megaureter (POM) may require timely intervention to prevent complications such as recurrent urinary tract infections or progressive renal dysfunction [9]. Advances in minimally invasive techniques, including PAUS, have provided effective and patient-specific treatment options for these conditions. However, the lack of a standardized system to classify dilated urinary ducts and guide treatment decisions remains a significant challenge [10,11].

In this research project, we set out to categorize urinary duct shapes with a method and assess its practical implications. We categorized the duct shapes into four groups as follows: shape; gently curved or straightened enlargement; “S”-shaped expansion; and spiral expansion. With this categorization in mind, we explored the impact of duct shapes on the procedure’s success rate and level of difficulty.

Our study focuses on demonstrating the effectiveness and safety of using imaging guidance for over-the-top stenting in children as evaluating the practicality of the newly devised classification system we created in clinical settings for assessing urinary duct shapes and comparing various treatment options in a standardized manner.

## 2. Materials and Methods

This study was planned retrospectively and approved by the Institutional Review Board. This study included pediatric patients aged 0–12 years referred for antegrade stenting after unsuccessful retrograde attempts between January 2011 and February 2024. Retrograde DJ stenting was initially attempted in all cases, and only those with retrograde failures underwent antegrade procedures. Data were obtained from the records of a tertiary research hospital. Cases were collected retrospectively from the PACS system and hospital database.

Data Collection and Analysis: Information was collected from images, procedure reports, patient notes and consultations. The following data were analyzed:

- Patient information;
- Reasons for diagnosis and treatment;
- Dimensions of the stent (thickness and length);
- Whether balloon dilatation was performed;
- Problems during or after the procedure;
- Reasons for stent removal and duration of stenting.

Each stent procedure was evaluated as a separate case. The success of the procedure was determined by comparing the results of ultrasound and MAG3 scintigraphy performed one month after stent removal with the pre-procedural results.

Purpose of Tc-99m MAG3 Scintigraphy: This test evaluates the functioning of the kidney and urine flow [12,13]. The tests performed before and after the procedure are compared to determine whether the stent is beneficial or not.

Technical Procedure: Patients were sedated and placed in a prone position. Cefixime was given as a prophylactic antibiotic. The procedure was performed by experienced radiologists under ultrasound and fluoroscopy guidance. The kidney was entered with a special needle set. Balloon dilatation was performed according to the stenosis. Special thin wires and catheters were used in S-shaped and spiral ureters.

**Figure 1:**
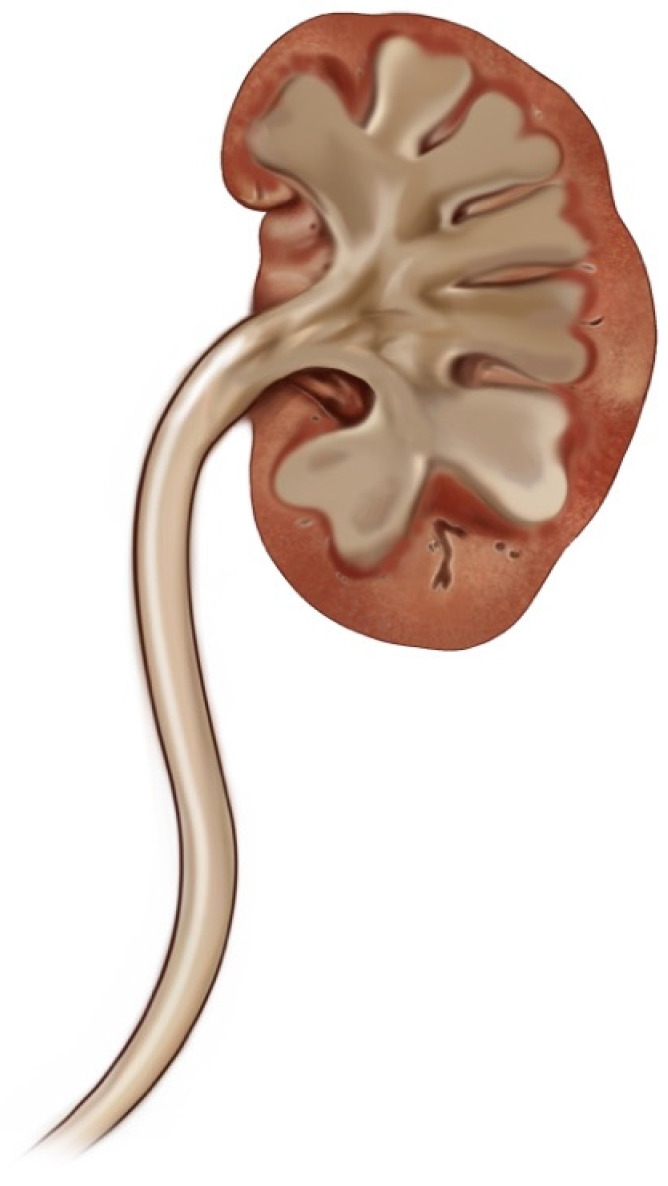
Category 0.

**Figure 2:**
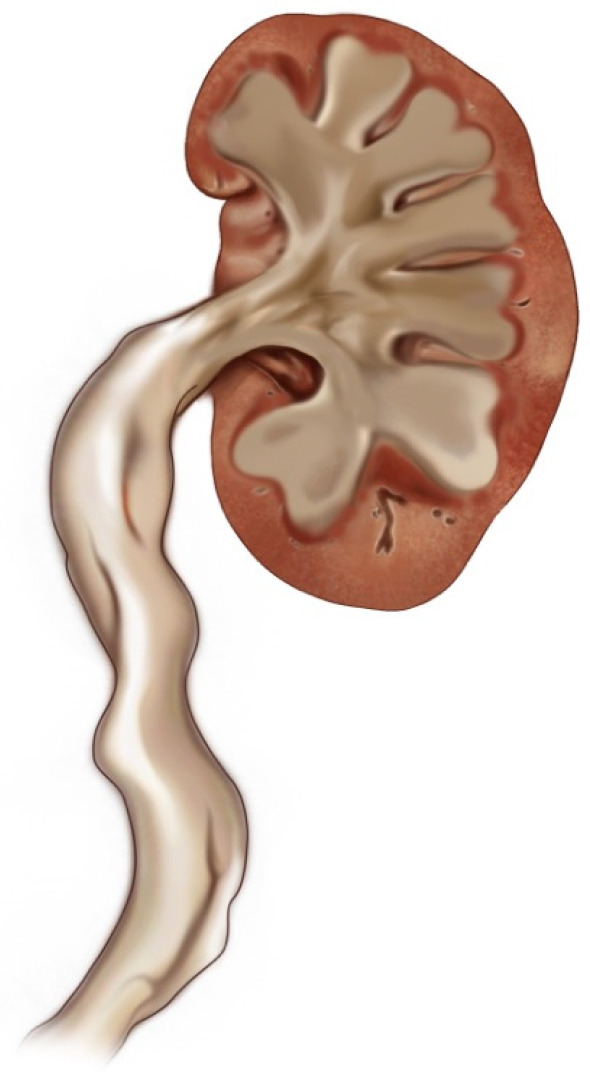
Category 1.

**Figure 3:**
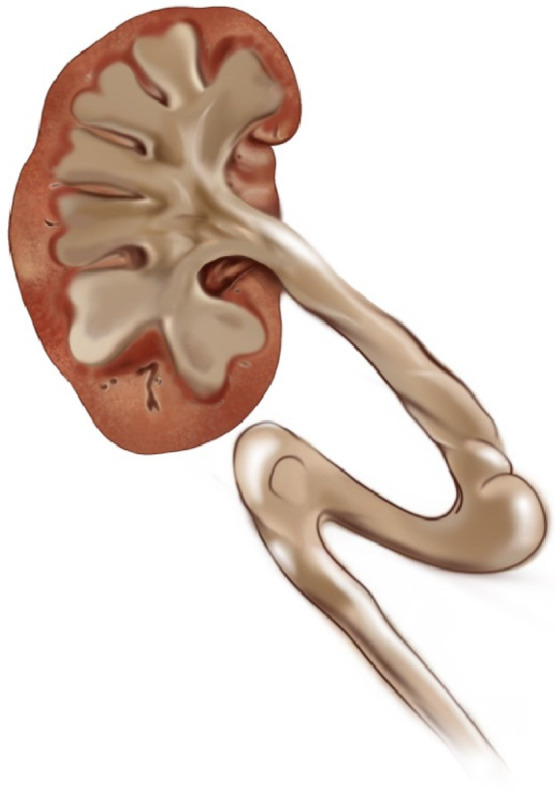
Category 2.

**Figure 4:**
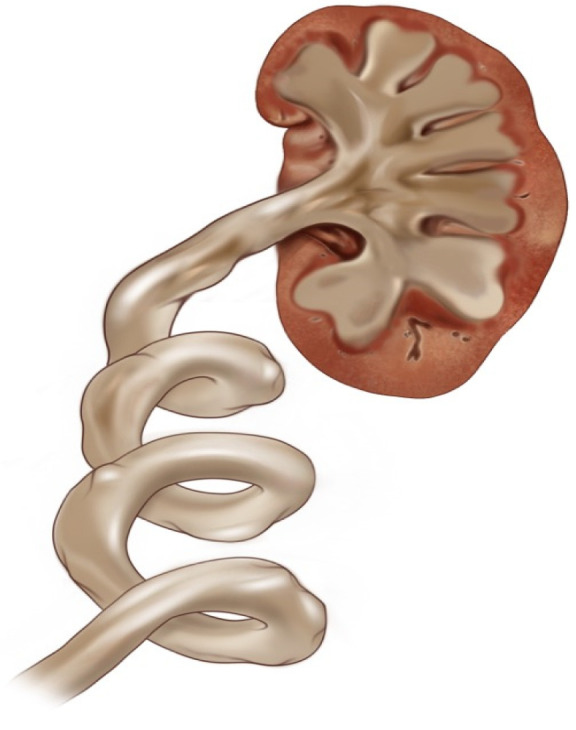
Category 3.

Hydroureter Classification and Hydronephrosis: In this study, we developed a new classification based on the shape and width of the ureter. We identified four main groups:

- Category 0: Normal ureter—(For example: “Normal ureter” refers to a ureter with a diameter of less than 6 mm, without any morphological findings suggestive of stricture or obstruction at the ureteropelvic junction). (Figure 1)
- Category 1: Straight or slightly curved—There is little expansion and slight curvature. (Figure 2)
- Category 2: S-shaped—There is a pronounced S-shaped curling and widening. (Figure 3)
- Category 3: Spiral-shaped—Advanced curling and expansion. (Figure 4)

Onen classification was used to evaluate the swelling in the kidney [14].

Success Criteria:

- Technical success: Correct placement of the stent.
- Functional success: Improvement in ultrasound and MAG3 tests.

Both Tc-99m MAG3 scintigraphy and ultrasound (USG) were utilized to evaluate changes before the procedure, as well as one month after stent removal, to assess both diagnostic and therapeutic outcomes. Scintigraphy was performed to monitor renal function and assess obstruction relief, while USG was used to evaluate changes in hydronephrosis. Post-procedural evaluations focused on identifying obstruction clearance, improvement in renal function, and partial or complete resolution of hydronephrosis.

Complications were divided into major and minor according to radiology standards [15]. Major complications were defined as conditions requiring treatment or hospitalization for more than two days. Minor complications were problems requiring short-term observation.

Statistical Analysis: Data were analyzed with SPSS V23. Normal distribution was evaluated by Shapiro–Wilk and Kolmogorov–Smirnov tests. Fisher–Freeman–Halton and Fisher’s Exact tests were used to compare groups. Mann–Whitney U test was used to compare non-normally distributed data. Spearman correlation test was used to examine the relationships. The significance level was set at *p* < 0.050.

Ethical Issues: This study was conducted in accordance with the ethical guidelines of the Declaration of Helsinki and was approved by the Başkent University Institutional Review Board (project no: KA21/228, date: 04.05.2021). Consent was obtained from the families or legal representatives of all patients.

## 3. Results

Descriptive Statistics: In the study, a total of 107 stent implantation procedures were performed in 71 pediatric patients and 76.6% of the patients were male. The mean age was 42.65 ± 47.31 months. The most common pathology was UVJ obstruction with a rate of 66.3% and stents were mostly placed on the left side (55.1%). The mean stent retention time was 96.34 ± 38.11 days. The technical success rate was 99.1% and the reason for stent removal was recorded as treatment termination in 87.8% of cases. The functional success rate was 84.1%. According to ureteral morphology, the most common structure observed was spiral shape with 41.1%. Details of descriptive statistics are presented in Table 1.

Complications observed during and after the procedure were classified as major and minor.

- Complications During the Procedure: During the procedure, 2.8% (*n* = 3) stent malposition, 0.9% (*n* = 1) perirenal hematoma, and 1.9% (*n* = 2) intra-pelvic-oral hematoma were observed. In cases of malposition, short stents were removed and replaced with longer stents. Other complications were monitored without additional intervention (Table 1).
- Post-Procedure Minor Complications: The most common minor complication was hematuria with a rate of 27.1% (*n* = 29). In patients with macroscopic hematuria, urine color returned to normal within the first 24 h and no additional intervention was required.
- Major Post-Procedure Complications: Stent migration (2.8%; *n* = 3) and fever due to urinary infection (8.4%; *n* = 9) were the main major complications. In cases of stent migration, the stent was removed and patients were re-evaluated. Patients with fever were treated with antibiotics, and the stent was removed early in resistant cases. There was no significant difference between intra-procedural complications and stent placement side (*p* = 0.794) and between post-procedural complications and balloon dilatation (*p* = 0.527). There was also no statistically. significant relationship between ureteral morphology and complication rates (*p* = 0.839) (Table 2).

**Table 2:** Surgical outcomes and procedural complications related to functional success and balloon dilation.

Question	Test (in Parentheses)	Test Statistic	*p*-Value
Did surgical operations impact functional success?	Mann–Whitney U Test (Surgical Operation and Functional Success)	12.50	1.000
Are intraoperative complications related to the side of stent placement?	Chi-Square Test (Intraoperative Complications and Side of Stent Placement)	3.12	0.794
Are postoperative complications associated with balloon dilation?	Chi-Square Test (Complications and Balloon Dilation)	4.16	0.527
Was ureter morphology associated with overall complication rates?	Chi-Square Test (Complications and Ureter Morphology)	9.68	0.839

**Figure 5:**
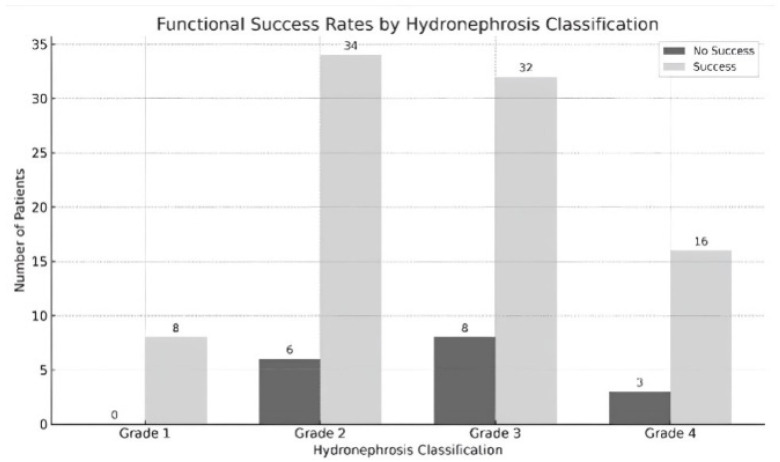
Functional success rates by hydronephrosis classification.

**Figure 6:**
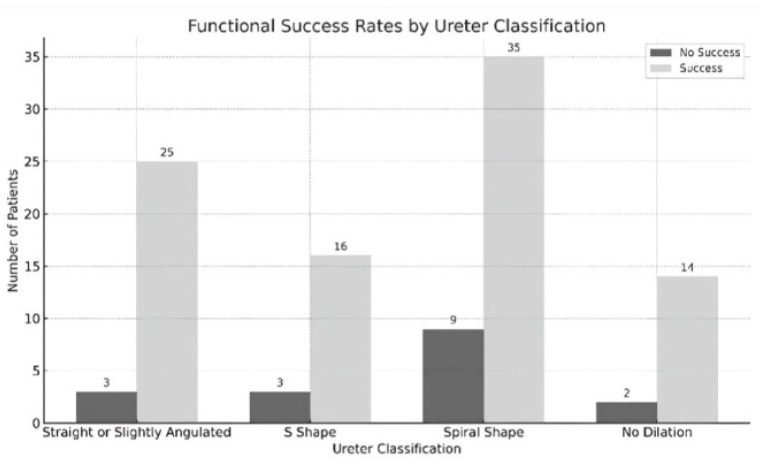
Functional success rates by ureter classification.

Follow-up and Evaluation of Results: The mean follow-up period was 96.34 ± 38.11 days and the technical and functional success rates were 99.1% and 84.1%, respectively. When the functional success rates were analyzed according to the degrees of hydronephrosis, a 100% success rate was obtained in Grade 1, 85% in Grade 2, 80% in Grade 3, and 84.2% in Grade 4. There was no significant difference between these groups (*p* = 0.721). In terms of ureteral morphology, functional success was 87.5% in normal ureter, 89.3% in straight or slightly angulated ureter, 84.2% in S-shaped ureter, and 79.5% in spiral-shaped ureter. There was no significant correlation between ureteral morphology and functional success (*p* = 0.775). There was no significant difference between functional success and stent diameter (*p* = 0.349), balloon dilatation (*p* = 0.927) or use of cutting balloon (*p* = 1.000) (Table 3, Figure 5 and Figure 6).

Post-procedural Tc-99m MAG3 scintigraphy revealed obstruction clearance and improvement in renal function in 84.1% of cases. Ultrasound follow-up, performed one month after stent removal, demonstrated partial or complete resolution of hydronephrosis in 89.2% of cases. Among these, 84.1% of patients showed both improvement in scintigraphy findings and partial or complete resolution of hydronephrosis on ultrasound. These combined findings highlight the effectiveness of the intervention in restoring renal function and alleviating hydronephrosis.

Functional Success and Underlying Pathology: Functional success was 80.8% in UPJ obstruction, 83.1% in UVJ obstruction, and 100% in ureterolithiasis, PUV, and transplanted renal ureteral stenosis. There was no significant difference between the underlying pathology and functional success (Table 3).

Surgical Operations and the Relationship between Functional Success and Complications: No significant relationship was found in the analyses performed to evaluate the effect of surgical interventions on functional success. This indicates that surgical procedures do not have a direct effect on functional success rates. When the relationship between the complications occurring during the procedure and the side of stent placement was analyzed, no significant relationship was found (*p* = 0.794). Similarly, there was no significant association between post-procedural complications and balloon dilatation (*p* = 0.527), indicating that balloon dilatation does not increase the risk of post-procedural complications (Table 2).

Results Related to Age and Other Factors: A moderate positive correlation was found between age and stent diameter (r = 0.509, *p* < 0.001), indicating that larger diameter stents were preferred with increasing age. There was also a significant correlation between age and the need for balloon dilatation (r = 0.490, *p* < 0.001). These data suggest that age may have an effect on technical preferences during stent implantation (Table 4).

In our study, antegrade ureteral stent placement had high technical (99.1%) and functional success (84.1%) rates. Complications were generally minor, with hematuria being the most common complication. There was no significant association between complications after stent placement and factors such as balloon dilatation or ureteral morphology. Underlying pathologies and surgical interventions were also found to have limited impact on functional success. These findings support the safety and efficacy of antegrade ureteral stent placement in pediatric patients.

## 4. Discussion

Our research focused on assessing how effective and safe it is to use imaging guidance for placing stents in children. Additionally, we created a classification system based on the morphology of the ureter. The results show that antegrade stent placement is an effective treatment method in the pediatric patient group with a high technical success rate (99.1%) and an acceptable complication rate (5.6%). The functional success rate was 84.1%, which is consistent with similar studies in the literature. The developed classification provided an objective evaluation of the effect of the morphologic features of the ureter on the stent placement procedure and the clinical applicability of this classification was emphasized. Functional success rate was found to be lower, especially in spiral-shaped ureters, but this difference was not statistically significant.

In the literature, two important studies on percutaneous antegrade ureteric stent placement have been conducted in the last 10 years. Herdem et al. [7] reported a technical success rate of 97.6% and a clinical success rate of 90%. Sertic et al. [16] reported a technical success rate of 95% and a functional success rate of 95%. In our study, the technical success rate was 99.1% and the functional success rate was 84.1%. These results show that our study offers the highest rate in terms of technical success. It is clearly supported that antegrade stent placement is a reliable method with high technical success rates.

Functional success, according to Sertic et al. [16], was the highest and was said to have consisted of urinary drainage and the decompression of the collecting system and was assessed on clinical, biochemical, and ultrasound levels. Herdem et al. [7] in their study, used the stent functioning and the absence of complications as criteria for clinical success. In the present study, methods like Tc-99m MAG3 scintigraphy and ultrasound were deployed to evaluate aspects of renal tissue enhancement and urinary flow improvement, thus suggesting broader evaluation of functional success in the present study than previously. If all three studies posit that high levels of technical success is attained in younger patients, it is paradoxical that each of the studies has a varying overall functional success evaluative criteria. This study has offered a new outlook in some ways on how functional success can be evaluated. In the particular case of functional success, the Tc-99m MAG3 scintigraphy used in this study provides an accurate technical evaluation. In other words, this technique determines whether renal function remains intact and whether there is an improvement in the flow of urine [10]. From this perspective, our study also makes an important step forward as it implements a strategy that integrates clinical perspective as well as demonstrating that there is functional recovery of the kidneys. Carrying out such evaluations on an objective basis, particularly among children, is likely to aid in the making of better therapeutic decisions.

Dean et al. [17] also reported that ureteral wall thickness (>3.2 mm) is an important criterion for predicting retrograde stent placement failure. Such morphologic assessments facilitate accurate treatment decisions and provide a more objective approach to patient management. A classification system based on ureteral morphology plays a similar role in predicting the success of antegrade stent placement procedures and may make important contributions to clinical practice. Similarly, in our study, functional success rates were found to be lower in spiral-shaped ureters. This finding reveals the effect of structural features of the ureter on the results of the procedure.

The reasons for technical failures in ureteral stent placement are based on various factors and these factors may affect the overall success of the procedure. One of the most important reasons for technical failures is anatomical difficulties. Tortuosity of the ureter, tight obstructions, and entrapment during stent placement are prominent [18]. In cases such as extrinsic compression, malignant tumors or retroperitoneal fibrosis, the ureters are subjected to external pressure, which also leads to improper stent placement. In such cases, alternative methods such as a percutaneous nephrostomy tube may need to be used in patients who cannot be stented [19]. Technical problems such as failure to place the stent in the correct position or failure to find the ureteral orifice are also common causes of failure [20]. Similarly, in our study, we observed increased technical difficulties in stent placement in spiral-shaped ureters. Especially the morphologic structure of the ureter may cause technical failures by making the correct positioning of the stent difficult.

Lee et al. [21] discussed other factors that may affect functional success in addition to stent diameter. In particular, the potential effects of patient age, reason for stent implantation, duration of stent implantation, and the level of ureteral stenosis on stent failure were evaluated. They found that stent diameter (especially 3-Fr stents) was the only significant risk factor for stent failure [21]. In our study, no significant correlation was found between stent diameter and functional success. This difference may be due to age differences in the patient population, the stent placement techniques used (antegrade vs. retrograde) or the effect of classification based on ureteral morphology. In addition, in our study, the effect of other factors such as ureteral morphology, degree of hydronephrosis, and use of balloon dilatation on functional success was examined, but no significant relationship was found. This suggests that the factors affecting functional success are multidimensional and not only dependent on stent diameter. In conclusion, to improve functional success in pediatric ureteral stenting procedures, other factors such as ureteral morphology and patient characteristics should be considered in addition to stent diameter.

Megaureter may create anatomical difficulties in stent placement and may lead to malpositioning [22]. Mao et al. [22] reported that megaureter may increase the risk of complications during stent placement. Especially in dilated or tortuous ureters, the difficulty of accurate stent placement makes the selection of the appropriate stent size even more critical [22]. In our study, malposition cases were observed in spiral-shaped and advanced tortuous ureters. This suggests that anatomical variations should be taken into account when placing a stent.

The complication rates observed in our study largely overlap with the results reported in the literature. Awad et al. [23] reported a complication rate of 41% after DJ stent placement and the most common complications were stent migration and infections [23]. In addition, Zhu et al. [24] reported that double-J stent placement after laparoscopic pyeloplasty in children was a safe method with a success rate of 96.96%, but complications such as stent migration, obstruction, and infection were reported to occur at a rate of 3.04% [24]. Noh et al. [25] reported a 90% success rate during antegrade stent placement, but complications such as urine leakage were observed in one case [25]. In order to prevent and manage complications, careful patient selection, optimization of techniques used during stent placement, and close monitoring of patients in the postoperative period are very important. Early removal of stents is recommended in the literature to reduce stent complications. Correct application of surgical techniques and regular follow-up in the postoperative period are critical to prevent stent migration and infections [24,25]. In addition, appropriate antibiotic prophylaxis and early intervention in cases with complications may be effective in reducing infection rates.

Our study focuses on short-term outcomes, particularly radiological and functional success assessed by Tc-99m MAG3 scintigraphy and ultrasound. However, clinical improvement parameters such as proteinuria, hypertension, urinary tract infections, and renal function changes require longer follow-up periods for meaningful evaluation. This is consistent with findings by Koyuncu et al. [26], who highlighted the need for close monitoring in patients with renal anomalies, and by Kim et al. [9], who emphasized integrating clinical parameters with radiological findings for a holistic assessment. Future studies should aim to incorporate extended follow-up durations and prospective designs to provide a more comprehensive evaluation of both radiological and clinical outcomes.

This research is the most extensive data on antegrade ureteric stenting in children. Restated limitations are also available. The backward-looking approach of some research might create gaps in data, therefore making some connections hard to establish. In addition, the single-institution practice narrows the scope of applicability of the findings, and a small sample can hinder economies of scale in the more sensitive determination of impact level of statistical tests. While the study sought to explore the extent to which the anatomy of the ureter impacts the degrees of difficulty of surgical procedure, the radiation doses and the time taken for the procedure were not explained. This limitation narrows down to the extent that morphologic features of difficulty can be described in terms of radiation exposure and time framework of a procedure. The next stage multicenter prospective interventional studies with these parameters included should be undertaken to triangulate the outcomes of the study and test more exhaustively the validity of the ureteral morphologic classification. Such data may provide significant inputs into the clinical decision-making processes with respect to patients’ safety and the efficacy of the procedures.

## 5. Conclusions

It was shown in this study that antegrade ureteric stenting can be safely and effectively performed on the pediatric population with acceptable rates of complications. In addition, the described ureteral morphologic classification complements the literature and enriches the understanding of the role played by ureteral morphology in the success of the procedure. Furthermore, this classification and the functional success criteria in this study can also be a reference for other studies and present a new direction for future research. The results endorse antegrade stent placement, particularly in cases where the retrograde approach is not feasible to be an effective alternative.

## Figures and Tables

**Table 1:** Descriptive statistics of variables.

Variable	Frequency (n)/Mean ± SD	Percentage (%)/Median (Min–Max)
Male	82	76.6
Female	25	23.4
Age (Months)	42.65 ± 47.31	24.00 (0.60–144.00)
Underlying Pathology		
UPJ Obstruction	26	24.3
UVJ Obstruction	71	66.3
Ureterolithiasis	4	3.7
PUV	3	2.8
Transplanted Kidney Ureter Stenosis	2	1.9
Leak in Right Ureter Lower End (Perioperative Complication)	1	0.9
Side of Stent Placement		
Right	42	39.3
Left	59	55.1
Transplanted Kidney	6	5.6
Duration of Stent Placement (Days)	96.34 ± 38.11	100.00 (15.00–210.00)
Stent Diameter (F)	6.18 ± 1.49	7.00 (3.00–8.00)
Stent Length	22.37 ± 4.94	26.00 (11.00–28.00)
Technical Success		
No	1	0.9
Yes	106	99.1
Procedure-Related Complications (Intraoperative)		
None	101	94.4
Stent Malposition	3	2.8
Perirenal Hematoma	1	0.9
Hematoma within Pelvicalyceal System	2	1.9
Post-Procedure Complications (Minor and Major)		
None	65	60.7
Fever	9	8.4
Stent Migration	3	2.8
Pain	1	0.9
Hematuria	29	27.1
Pre-Stent Surgical Procedures		
None	54	50.5
Pyeloplasty	11	10.3
Vesicostomy	2	1.9
PUV Ablation	15	14.0
Operated VUR	4	3.7
Ureteroneocystostomy	16	15.0
Vesicostomy	1	0.9
Ureterorenoscopy	2	1.9
Urinary Diversion	2	1.9
Post-Stent Surgical Procedures		
None	103	96.3
Yes	4	3.7
Reason for Stent Removal		
Treatment Completed	94	87.8
Infection	4	3.7
Stent Migration	3	2.8
Pain or Discomfort	1	0.9
Catheter Dysfunction	5	4.7
Functional Success		
No	17	15.9
Yes	90	84.1
Balloon Dilation		
Not Performed	43	40.2
Performed	64	59.8
Balloon Dilation Size Before Stent Placement (diameter × length) (mm)	207.19 ± 87.48	200.00 (80.00–480.00)
Hydronephrosis (Grade)		
Grade 1	8	7.5
Grade 2	40	37.4
Grade 3	40	37.4
Grade 4	19	17.8
Ureter Classification (Straight or Slightly Angulated, S-shape, Spiral Shape)		
Straight or Slightly Angulated	28	26.2
S-shape	19	17.8
Spiral Shape	44	41.1
No Dilation	16	15.0

Notes: UPJ: ureteropelvic junction; VUR: vesicoureteral reflux; UVJ: ureterovesical junction; PUV: posterior urethral valve; F: French (diameter unit for catheters); Grade: hydronephrosis grading.

**Table 3:** Distribution of functional Success by hydronephrosis, ureter classification, and underlying pathology variables.

Variable	Functional Success	Total (n, %)	Test Statistic *	*p*-Value
	No (%)	Yes (%)		
Hydronephrosis (Grade)				
Grade 1	0 (0)	8 (100)	8 (7.5)	1.588
Grade 2	6 (15)	34 (85)	40 (37.4)	
Grade 3	8 (20)	32 (80)	40 (37.4)	
Grade 4	3 (15.8)	16 (84.2)	19 (17.8)	
Ureter Classification				1.271
Straight or Slightly Angulated	3 (10.7)	25 (89.3)	28 (26.2)	
S-Shape	3 (15.8)	16 (84.2)	19 (17.8)	
Spiral Shape	9 (20.5)	35 (79.5)	44 (41.1)	
No Dilation (Normal Ureter)	2 (12.5)	14 (87.5)	16 (15.0)	
Underlying Pathology			---	---
UPJ Obstruction	5 (19.2)	21 (80.8)	26 (24.3)	
UVJ Obstruction	12 (16.9)	59 (83.1)	71 (66.3)	
Ureterolithiasis	0 (0)	4 (100)	4 (3.7)	
PUV	0 (0)	3 (100)	3 (2.8)	
Transplanted Kidney Ureter Stenosis	0 (0)	2 (100)	2 (1.9)	
Leak in Right Ureter Lower End	0 (0)	1 (100)	1 (0.9)	

Notes: * Fisher–Freeman–Halton test applied; values presented as frequency (percentage); suitable for distribution comparison; ureter classification is a unique classification system developed specifically for this study.

**Table 4:** Comparison of functional success by stent diameter, balloon dilation, age, and cutting balloon use.

Variable	No Functional Success (*n* = 11)	Functional Success (*n* = 53)	Test Statistic (*p*-Value)
Stent Diameter (F)	6.55 ± 1.23	6.11 ± 1.53	658.5 (0.349)
	Median: 7.00 (Range: 4.00–8.00)	Median: 7.00 (Range: 3.00–8.00)	
Balloon Dilation Before Stent Placement	5.36 ± 0.92	5.38 ± 1.15	286.5 (0.927)
	Median: 5.00 (Range: 4.00–7.00)	Median: 5.00 (Range: 4.00–8.00)	
Cutting Balloon Use			
No	10 (18.2%)	45 (81.8%)	1.000
Yes	1 (11.1%)	8 (88.9%)	
Correlation Coefficient (r)			
Age with Stent Diameter (F)	0.509		<0.001
Age with Balloon Dilation	0.490		<0.001

Notes: F: French (diameter unit for catheters); test values are presented as mean ± SD and median (min–max) for continuous variables; percentages for categorical variables.

## Data Availability

The data supporting the reported results of this study are not publicly available due to privacy and ethical restrictions. However, de-identified data may be made available from the corresponding author upon reasonable request and with permission from the Institutional Review Board of Başkent University.
